# Structural and electronic properties of AgNPs adsorbed by glucose molecules determined using DFT theory

**DOI:** 10.1016/j.heliyon.2024.e38890

**Published:** 2024-10-02

**Authors:** Walaa S. Sarhan, Nagham M. Shiltagh

**Affiliations:** Department of Physics, College of Science, University of Kerbala, Karbala, Iraq

**Keywords:** Silver nanoparticles, Glucose, Adsorption, IR spectrum, HOMO and LUMO, DFT

## Abstract

In order to build and design stable molecular structures of α‐D‐glucose molecules after adding to a cluster of silver atoms, an optimization process was precisely carried out for α‐D glucose/Ag3 molecule at low energy. The correlation between glucose molecule and silver atoms is evaluated by investigating configurational and electronic features of the named molecules by adopting the Density functional theory DFT using the hybrid B3LYP functional and 6–311+G∗ as a basis set for C, O, and H atoms, while LANL2DZ set for silver (Ag) atoms. Frequencies of vibrational modes are essential analyses that have been instrumental in understanding the IR spectra of studied molecules. These analyses enable the detection of the active groups along the spectrum chart including C–O–C, C=O, C–O, O–H, C–C and C–H peaks confirming the previous experimental findings. Another important finding is the energy gap (E***g***) obtained by the difference between the higher occupied molecular orbital (HOMO) and lower unoccupied molecular orbital (LUMO). Remarkably, E***g*** was found to increase from 3.440 to 4.358 eV in a configuration consisting of two glucose molecules with one Ag atom (2α‐D glucose/Ag-C_12_H_24_O_12_Ag) to another with one glucose molecule with three Ag atoms (α‐D glucose/Ag3-C_6_H_12_O_6_Ag_3_) configuration. Additionally, the potential of the molecular electrostatic isosurface (MEP) in 3D diagram for two configurations is clarified by the colour-coded bar with the distributions of charge density distributions to examine the nucleophilicity and electrophilicity behaviour.

## 1 Introduction

Nanotechnology is currently one of the fastest-growing fields due to its multiple applications. Metal nanoparticles have chemical and physical properties, in particular. It has been used in a wide range of applications, including medication administration, amino acids, and antimicr-obials, and shows some promise in cancer diagnosis and treatment [1,2]. Silver nanoparticles (AgNPs) are one of the most remarkable examples of metal nanoparticles that have stimulated research interest [3,4]. Therefore, AgNPs have been utilized in a diversity of applications, including in the medicine, food, health, and cosmetics industries and this is due to their remarkable characterizations in physics and chemistry [5,6]. The properties of nanoparticle surfaces are determined by the synthesis process utilized. Absorption and fluores-cence spectra are important tools in the analysis of spectral lines and optical properties of atoms and molecules [7], and have been employed to study the features of silver nanoparticles prepared via various chemical and physical methods [8,9]. The preparation method of silver nanoparticles with α‐D‐glucose and D‐gluconate adsorption on surfaces as capping agents was investigated by Gonzalez et al. experimentally and theoretically (DFT) via Raman spectroscopy [10]. Hybrid nanopar-ticles, comprised of two or more types of separate nanocomponents, are emerging that can improve the characteristics of various nanomaterials or create a synergistic impact. For instance, organic compounds or AgNPs in inorganic compounds that have been employed to extinct the chemical surfaces or modify chemical compounds, are appropri-ate factors by Cdots investigations. Furthermore, the determination of glucose levels in human tissues by the ineffective surface could enhance the physical properties such as fluorescence factor [11]. In recent years, in order to limit the use of hazardous reagents such as NaBH_4_, novel synthesis techniques based on natural reagents, such as glucose, have been developed [12]. Various mechanisms can be used to stabilize colloids, such as electros-tatics (a), steric effects (b), hydration forces (c), depletion forces (d), and van der Waals forces [8,13]. The effectiveness of AgNPs is significantly correlated to the molecules adsorbed on their surface. A strong evidence of antibacterial characteristics is demonstr-ated by Ref. [14] between, either NPs adsorbed by (glucose or galactose) or adsorbed by (maltose or lactose), however, NPs with disaccharides demonstrate effective antibac-terial properties. The shape, size, and Z potential of several capping agents such as polyethylene glycol, ethylenediaminet-etraacetic acid, polyvinylpyrrolidone, and polyvinyl alcohol were also examined [15]. These syntheses were employed to explore the mechanism of coated nanomagnetite synthesis from a single Fe (III) precursor in sucrose, where the sucrose functions as a bifunctional agent in two ways: (i) it decomposes into reducing species, causing partial reduction of the Fe^3+^ ions to Fe^2+^ ions, which is required for the formation of Fe_3_O_4_, and (ii) it acts as a capping agent source, adjusting surface properties and allowing the formation of nanoscale particles [16]. Furthermore, Peng et al. used bamboo hemicelluloses as a stabilizer and glucose as a reducer, and a simple green microwave-assisted technique of manufact-uring silver nanoparticles was devised in an aqueous medium. The manufacture of silver nanoparticles was quick and environment-tally friendly [17]. On the other hand, theoretical investigations have been frequently utilized to estimate features that are difficult to study experimentally. Density Functional Theory (DFT) is one of the various sophisticated methods that enable building and design variety of maters compounds through the determination of geometrical structures [18]. In addition, computational processes can provide precise scales in chemical interactions, electronic structure and combinations processes, where several theoretical calculations via DFT have been relied on or otherwise taken into account in practical applications [19]. Although a previous study demonstrated the use of SERS and DFT calculations to present amino acid absorption on silver nanoparticle surfaces. Hence, chemisorption has been obtained strongly via atoms of nitrogen and oxygen of the adsorbent surface with silver particles [20]. New approaches for characterizing the geometries of nanoparticles created by new synthetic procedures are urgently needed. Another study employed SERS and DFT to determine multiple structures of the Ag clusters with glucose, analyzing vibrational frequencies and Raman spectra [10], demonstrating the possible adsorption geometries of D‐gluconate and D‐glucose on the silver surface. However, which is the condition of the connection of mentioned glucose structures with AgNPs more stable? Further, electronic properties are not indicated.

This work aims to provide a theoretical DFT-based analysis of the influence of nanomolecules (AgNPs) on the various possible structures of glucose molecules. Therefore, this work could well represent a promising investigation into the provision of a satisfactory approximation of the configu-ration of Ag clusters with glucose at the preferential adsorption geometry. The reason for selecting this scenario is to build the glucose/AgNPs composite with a suitable and stable configuration at nano behaviours for use in different applications.

## 2 | methods

### 2.1 Computational details

**Fig. 1:**
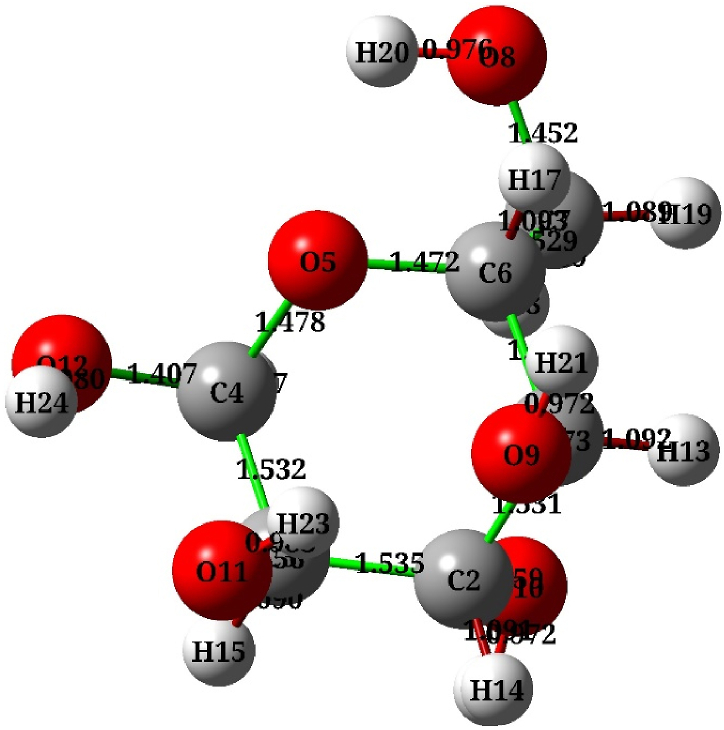
Optimized structure of α‐D‐glucose (C_6_H_12_O_6_) molecule with the corresponding oxygen atoms (in red), carbon and hydrogen atoms in grey and white, respectively, with bond length between atoms indicated.

Computational analyses have been used by various disciplines in physics and chemistry to gain an understanding of the molecular and electronic structure of chemical systems. A qualitative methodology is employed in the current study by adopting a hybrid function of B3LYP (Becke, three-parameters, Lee-Yang-Parr) and a basis set of Pople 6–311+G∗ set for C, O, and H atoms involved in the DFT theory for C, O, and H atoms [21]. However, LANL2DZ set was used for silver (Ag) atoms, [22]. Additionally, the inferred hydration instruction polarizable conductor calculation model (CPCM) was used to simulate the solvent effect. All those calculations and functions were conducted using the Gaussian 09 and GaussView 6.0 software [23]. The optimized molecules geometrically are considered a primary step of getting a stable molecule with lower convergence thresholds of energy. Therefore, a geometric optimization for isolated α‐D‐glucose structure (C_6_H_12_O_6_) was carried out, as exemplified in Fig. (1), to find stable molecules at lower energy. Silver nanoparticles (AgNPs) using one atom and a cluster of three silver atoms (3Ag) were also optimized, where D‐glucose adsorption geometries can be estimated theoretically on the silver surface. Consequently, these configurations suggest that the utilized silver cluster may be considered a satisfactory approximation for AgNPs. This cluster has been employed in a number of research efforts, including the analysis of IR spectra and electronic and thermal properties of the associated species. It was found to be an appropriate model for AgNPs, giving in a remarkable correspond-ence between experimental and theoretical results in the literature [24,25]. Two groups of molecular structures were assessed in this research; one of these configurations is related to 2 D-glucose with one Ag atom, whilst the second includes D-glucose with a cluster of three Ag atoms (closed triangular from Ag_3_), as will be clarified further in the following section.

## 3 Results

### 3.1 Molecular geometric optimization

Convergence criteria for the potential energy surface will be set up initially depending on parameters such as maximum forces, root-mean-square (RMS) forces, maximum displacement, and RMS displac-ement which must equal zero for small molecular structures, or minimal values for large molecules in a computational sense [24]. Therefore, after the optimization of the glucose molecules connecting to the silver nanoparticles, the resultant structures were optimized geometrically using tight conver-gence parameters. The values of optimized parameters are represented in Table (1), where these values for the 2α–D glucose/Ag configuration are 2 × 10^−6^, 0, 4 × 10^−5^, and 1.2 × 10^−5^, whereas for the α D-glucose/3Ag structure are 0, 0, 3 × 10^−6^, and 1 × 10^−6^.

**Table 1:** Optimized parameters at lower energy of two stable configurations for mentioned structures.

Structures	C_12_H_24_O_12_Ag	C_6_H_12_O_6_Ag_3_
Optimized Parameters		
Maximum Force	2 × 10^−6^	0
RMS Force	0	0
Max Displacement	4 × 10^−5^	3 × 10^−6^
RMS Displacement	1.2 × 10^−5^	1 × 10^−6^

**Fig. 2:**
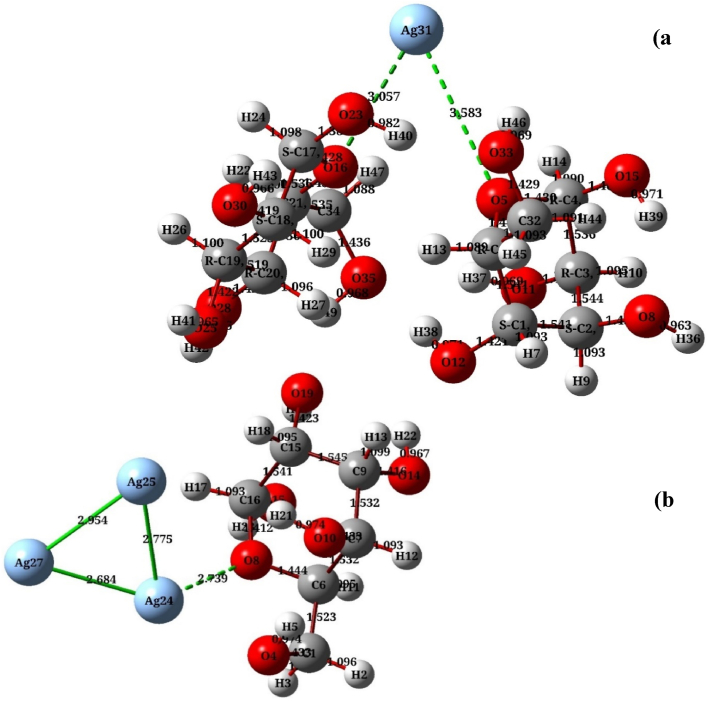
Final adsorption geometries for the α‐D‐glucose with Ag system: (a) 2 α – D glucose structure with 1-AgNPs, C_12_H_24_O_12_Ag (b) α-D-glucose structure with 3-AgNPs, C_6_H_12_O_6_Ag_3_. Oxygen, carbon, and hydrogen atoms are shown in red, grey, and white, respectively, whereas light blue atoms correspond to the silver atoms in the associated clusters. The two configurations are stable form, after optimization via DFT using B3LYP//6-311+G∗/LANL2DZ.

Fig. (2) Illustrates the final geometries for the above-mentioned configurations of the α‐D‐glucose molecule with Ag atoms: where (a) represents the configuration of 2α–D glucose with one silver atom – 1AgNPs (C_12_H_24_O_12_Ag), and (b) indicates to the α–D glucose with three silver atoms/3AgNPs (C_6_H_12_O_6_Ag_3_) in their most stable forms, after geometric optimization at low energy.

Selected geometrical parameters that were considered the most important structural dimensions of the above-mentioned config-urations are listed in Table (2). This geometrical analysis reports, in particular, the distance between the oxygen atom (O_8_) and silver atom (Ag_24_) for α-D-glucose-Ag_3_, which is always greater than 2.5 Å for the closed cluster (triangular form). For the second structure, 2α-D-glucose-Ag, on the other hand, the distance between the silver (Ag_31_) and oxygen (O_23_ and O_33_) atoms is around 3–3.58 Å. Consequently, in these cases, the adsorbate–Ag atoms interaction is fragile. Table (2) also presents bond length in Angstroms, the angle θ (^O^) between the three atoms in these configurations at the effective sites, and Electronic Energy.

**Table 2:** Dimensional coordinates for α‐D‐glucose on the Ag atom and Ag_3_ cluster including bond lengths, and bond angles in addition to the electronic energy.

Compound	Electronic Energy/eV	Assignment	Bond length/Å	Assignment	Angle θ/(^O^)
α-D-glucose–Ag3	−30604.934	Ag24-O8	2.739	Ag25–Ag24-O8	96.904
		Ag24–Ag25	2.775	Ag24–Ag25–Ag27	55.776
		Ag24–Ag27	2.648	Ag25–Ag27–Ag24	58.741
		Ag25–Ag27	2.954	Ag27–Ag24–Ag25	65.483
		C–C	1.54	Ag24-O8–C6	110.903
		O–H isolated	0.96	C–C–C (ring)	109–114
		C–O	1.44	C–O–C (ring)	115.498
		C–O isolated	1.43	C–O–H	107–111
2α-D-glucose–Ag	−41373.511	Ag31–O16	3.057	O5–Ag31–O16	56.243
		Ag31–O5	3.583	Ag31–O5–C4	113.487
		C–C	1.54	Ag31–O16–C21	124.567
		C–O	1.43	C–C–C	109–113
		C–H	1.08	C–O–H	107–111
		O–H	1.41	C–C–H	107–111
α-D-glucose (pure)	−30605.013	C–C	1.53	C1–C2–C3	112.431
		C–O	1.46	C2–O10–H22	110.848
		O–H	0.97	C4–O5–H6	116.412
		C–H	1.00	C6–O1–H3	110.332

### 3.2 Vibrational frequencies and IR spectra analysis

Under a minimum geometric optimization of studied structures, B3LYP//LANL2DZ function were employed to estimate vibrational harmonic frequencies. It was obtained 75 vibrational modes for one glucose molecule bound to a cluster of three silver atoms (C_6_H_12_O_6_Ag_3_) on the authority of equations (3N)-(6) for non-linear molecules, where N represents the number of atoms. On the other hand, the number of vibrational modes of the second configuration involving one Ag atom connecting to two glucose molecules (C_12_H_24_O_12_Ag) is 141 mode. Table (3) lists the main findings with regard to vibrational frequencies (in cm^−1^) of the α*‐*D-glucose molecules and Ag cluster. It was observed that there was a decrease in frequencies for the C_12_H_24_O_12_Ag structure. This is because the frequency is proportional inversely to the mass of the structure, according to the relation:

**Table 3:** Frequencies of α‐D‐glucose molecules connecting to Ag cluster obtained by DFT calculations for the normal vibrational modes.

Mode Number	Frequency *ν*/cm^−1^ (C_12_H_24_O_12_Ag)	Frequency *ν*/cm^−1^ (C_6_H_12_O_6_Ag_3_)
4	36.9500	38.6900
11	120.360	161.300
12	127.240	172.010
15	166.660	253.130
21	228.740	383.850
24	284.350	475.780
29	324.310	694.780
31	368.110	763.340
34	703.690	885.460
52	714.280	1361.26
53	880.440	1370.12
57	1009.32	1410.17
63	1019.56	1506.53
64	1026.21	3005.94
69	1072.85	3087.58
71	1087.29	3577.13
72	1092.56	3615.40
75	1111.97	3791.42
118	2985.29	–
119	2992.34	–
135	3690.83	–
138	3760.72	–

*v* = 1/2*π* √*k*/*μ*

IR spectra of glucose molecules with AgNPs were calculated and analyzed using a B3LYP//6–311+G∗/LANL2DZ model chemi-stry in the range 0–4000 cm^−1^. Several configurations were suggested to investigate how the interactions of silver with glucose impact the typical vibrational modes of various functional groups, where the latter act as capping agents on silver nanoparticle 1is the assignment of the frequencies to the various vibrational modes of the appropriate structures by comparison with the calculated harmonic vibrational frequencies at the B3LYP//LANL2DZ level.

**Fig. 3:**
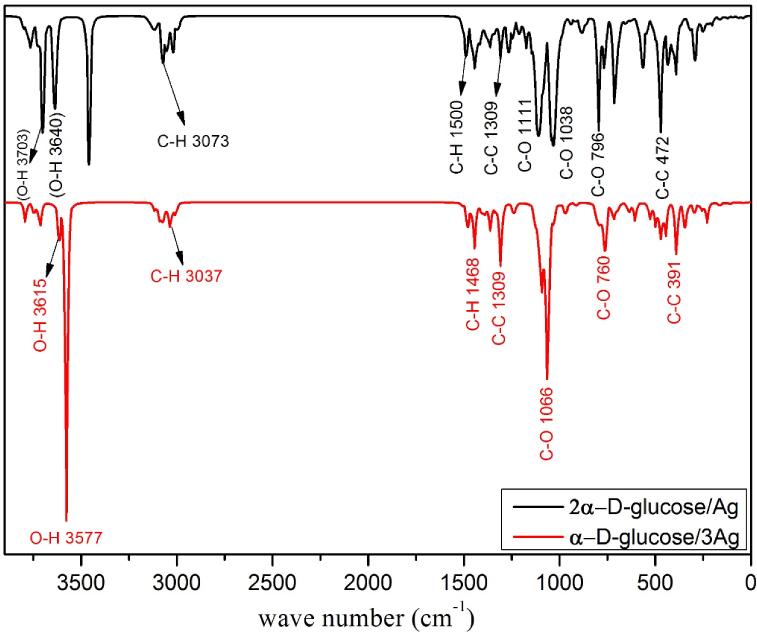
IR spectra of the two glucose structures with AgNPs; α-D-glucose-Ag_3_ (red), and 2 α-D-glucose-Ag (black), including the assignments of the most influential groups.

A comparison of the vibrational assignment of the normal modes, which is based on an analysis with a comparable functional group, descriptions for studied configure-ations are given in Table (4). Generally, it is observed from the analysis of IR spectra's that the distinctive bands due to the C-O and C-C groups of carbohydrates are in the range 400–1500 cm^−1^, with the C-H and O-H bands typically observed around 2900-3700 cm^−1^ [[26], [27], [28]]. The oxygenated functional groups were taken into consideration in both in terms of whether they interact directly with the silver atom(s) or otherwise. The assignment of the most influential groups is shown in Figure (3). These vibrational frequencies were found to match those in the literature for these species, indicating the possibility of adsorbed D-glucose on the silver surface, although there are a few minor differences that can be explained by the sample's varied carbohydrate concent-ration from that used in these other experiments [29,30]. Furthermore, it was observed that there were remarkable red shifts in the frequencies of the strong bands occurring at 3703 and 3460 cm^−1^ for α–D glucose/Ag_3_, which appeared at 3615 (O4–H5) and 3577 (O10-H21) cm^−1^, with respect to the 2α–D glucose/Ag structure. This indicates that the C–H bond is affected by the interaction of 2α–D glucose with the AgNPs cluster. “Red shift”, in this instance, indicates that the IR spectrum of α–D-glucose configuration with a closed cluster of Ag_3_ shifts towards shorter wavelengths (higher energies), confirming the effect of glucose, which acts as a capping agent [31].

**Table 4:** Comparison of calculated wavenumbers (cm^−1^) for final adsorption geometries of studied molecules indicating the vibrational normal modes of the main functional groups.

Molecular structure	Most probable assignment	Type of Mode	Approximate wavenumbers/cm^−1^
	*ν* C-O	side stretch,	760
	δ CCO, OCO	bending	
α-D-glucose/Ag_3_	*ν* C-O, C-C	Stretching,	1066
	δ COH	Bending	1039
	*ν* C-C	Stretching	1309
	δ (ρ_S_) C-H	Bending (sci.)	1468
	*ν* C-H	Stretching (sym.)	3037
	*ν* O-H	Stretching	3577
2α-D-glucose/Ag	*ν* C-O	side stretch,	796
	δ CCO, OCO	bending	
	*ν* C-O, C-C	Stretching,	1038
	δ COH	Bending	1111
	*ν* C-C	Stretching	1309
	δ (ρ_S_) C-H	Bending (sci.)	1500
	*ν* C-H	Stretching	3073
	*ν* C-H	Stretching (asym.)	3148
	*ν* O-H	Stretching	3640

*ν* = stretching, δ = bending, ρ_S_ = Scissoring is change in angle between a group of atoms in the same plane.

Sym. = symmetric, asym. = asymmetric, w = weak, s = strong, m = moderate, w = weak.

Additionally, the band at 796 cm^−1^ appears in the spectrum of the α-D-glucose/Ag structure, suggesting a dependence on the C-O configuration. This is also confirmed by the frequency shift to 760 cm^−1^ in the spectrum of α-D-glucose/Ag_3_. Analysis of normal coordinates [32] has shown that the CCO, OCO bending, and C-O stretching modes contribute to the weak band at 760 cm^−1^.

On the other hand, the 70-300 cm^−1^ frequency range also contains several interesting bands that were recognized through the animation in the GaussView program for the α–D glucose/Ag3 structure. Most of the bands appearing in this region arise from normal mode vibrations involving motions of Ag(24), Ag(25) and Ag(27). The bands at 71, 127, and 229 cm^−1^ include the Ag(24)- Ag(25)-Ag(27) bending (sci.), Ag(24)-Ag(25)-Ag(27) stretching (symm.), and Ag(24)-Ag(25)-Ag(27) stretc-hing (w) motions. However, the motion of AgNPs in the 2α–D glucose/Ag structure was detected at bands 94, 202, and 247 cm^−1^ involving the O(5)- Ag(31)- O(16) bending (sci.), O-Ag-O stretching (asymm.), and O-Ag-O stretching (mw) motions, respect-tively.

Another comparison between experimental and calculated vibrational frequencies (in cm^−1^) from previous studies that revealed a significant correlation is presented in Table (5).

**Table 5:** Comparison of experimental and theoretical wavenumbers for the currently studied molecules and previous studies.

Frequency/cm^−1^ (current study)		Theoretical	Experimental
2α-D-glucose/Ag	α-D-glucose/Ag_3_	Wavenumbers/cm^−1^ [33,34]	Wavenumbers/cm^−1^ [10,33]
391	391	395	▬
472	445	435	446
562	526	552	531
796	760	751	760–754
886	**▬**	850	806
940	967	991	996–942
1038	1039	1039 - 1032	1039
▬	1093	1090	1095 - 1094
1111	▬	1110 - 1103	1108
1174	▬	1170	1176
1246	1237	1254	1245
1264	▬	1292 - 1265	1284
1309	1309	1305	1307
1363	1363	1368	1363 - 1366
1444	1444	1440	1433 - 1425
1500	1480	1493	1450 - 1494
2992	3001	2957	2958
3073	3037	3072	3068
3460	3577	3508	3510

### 3.3 HOMO–LUMO gap

**Fig. 4:**
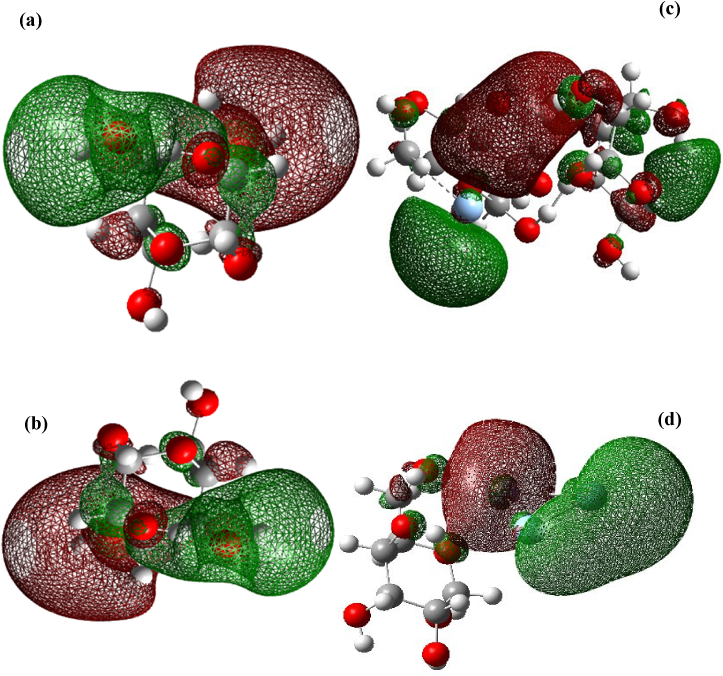
The frontier orbitals of the HOMO and LUMO state isosurfaces for different glucose molecules' adsorption onto silver nanoparticle surfaces. (a) is HOMO of glucose 2α–D glucose prior to adsorption on the one sliver atom, (b) is HOMO of glucoseα–D glucose with cluster of three silver, (c) represents LUMO of 2α–D glucose after adsorption with one silver atom, and (d) represents the adsorption of three silver clusters by α–D glucose.

The frontier molecular orbital (FMO) theory states that the chemical reactivity depends on how the HOMO and LUMO levels of the reacting species interact [35,36]. EHOMO is a quantum chemical parameter that refers to a molecule's capacity to donate electrons. The tendency of the molecule to donate electrons to an appropriate acceptor molecule with a low empty molecular orbital energy is likely indicated by a high EHOMO [36]. The molecular structure's capacity to take electrons is shown by the energy of the lowest unoccupied molecular orbital, or ELUMO [37]. As a result, the molecule takes electrons more readily the lower the value of ELUMO. With rising HOMO and falling LUMO energies, the inhibitor's ability to attach to the metal surface increases [38]. The frontier orbitals of adsorbed α-D-glucose on the silver cluster surface are illustrated in Fig. (4).

From Fig. 4(a and b), it is observed that HOMO states distributed on C and O atoms which resulted fundamentally from π molecular orbitals on C atoms of the carbon rings. However, LUMO states are raised principally from C π∗ molecular orbitals. On the other hand, after adsorption, HOMO states concentrate on the contact area between α-D-glucose and the Ag atom or Ag_3_ cluster interaction as shown in (a and b), where (a) represents HOMO for 2α–D glucose after adsorption with one silver atom, and (b) represents HOMO for the adsorption of three silver clusters by α–D glucose. However, LUMO states are totally sited on the silver cluster, not on the glucose molecule where (c) represents LUMO for 2α–D glucose after adsorption with one silver atom, and (d) represents LUMO for the adsorption of three silver clusters by α–D glucose. Consequently, increased charge transfer occurs following the interaction of D-glucose with silver. Finally, α-D-glucose HOMO and LUMO states that are distributed throughout the molecule become strongly localized on the O and Ag atoms following adsorption.

The calculated energy gap increases from 3.440 to 4.358 eV in a configuration consisting of two glucose molecules with one Ag atom (2α‐D glucose/Ag-C_12_H_24_O_12_Ag) to another with one glucose molecule with three Ag atoms (α‐D glucose/Ag_3_-C_6_H_12_O_6_Ag_3_) configuration as presented in Table 6.

**Table 6:** HOMO–LUMO gap interaction of AgNPs with α–D glucose as found via DFT theory.

Structure	ELUMO (eV)	EHOMO (eV)	LUMO–HOMO gap (eV)
C_12_H_24_O_12_Ag	**−0.718**	**−4.158**	**3.440**
C_6_H_12_O_6_Ag_3_	**−0.308**	**−4.666**	**4.358**

### 3.4 Molecular electrostatic potential (MEP) surface

**Fig. 5:**
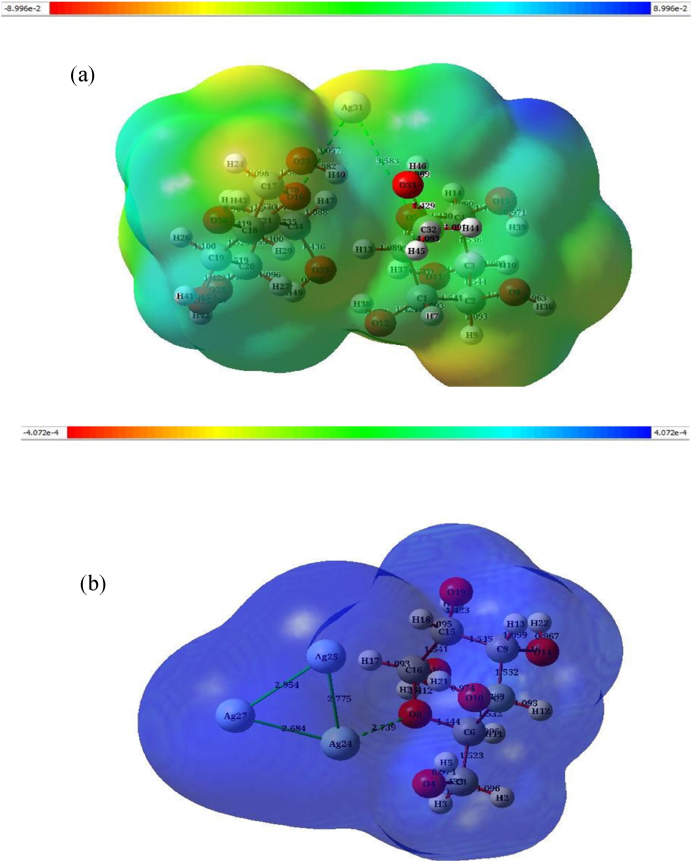
MEP isosurface surfaces diagram of the molecular electrostatic potential, in 3D, for (a) 2α-D glucose with 1 AgNP, and (b) α-D glucose with 3AgNPs, with the charge density distributions shown on the isosurface. Color-coded scales are shown above each figure.

Another finding that stands out from the results reported in this research is the molecular electrostatic potential (MEP) diagram reactions and densities of the electron with charge. This diagram was plotted to assess the molecules interaction through analysis of the intermolecular distance, nonlocalized responses of the structure and repulsive or attractive force between atoms. The red and blue areas indicate electron-rich and electron-poor regions, while the green color represents the neutral electrostatic potential. Fig. 5 (a and b) illustrates regions interaction by coloured lines indicating the main two zones along the upper edge of the figure in the range from −8.996 × 10^−2^ (red) to 8.996 × 10^−2^ (blue) for 2α-D-glucose with 1AgNPs, and −4.072 × 10^−2^ (red) to 4.072 × 10^−2^ (blue) for α-D-glucose with 3AgNPs, respectively. Also, there are multiple possible locations for electrophilic attack over the Ag31, Ag24, Ag25 and Ag27 atoms. Negative (red) MEP areas are related to electrophilic reactivity, whereas positive (blue) MEP regions have been linked with nucleophilic reactivity. Although areas with a negative potential are over electronegative atoms, areas with a positive potential are above hydrogen atoms [39].

**Fig. 6:**
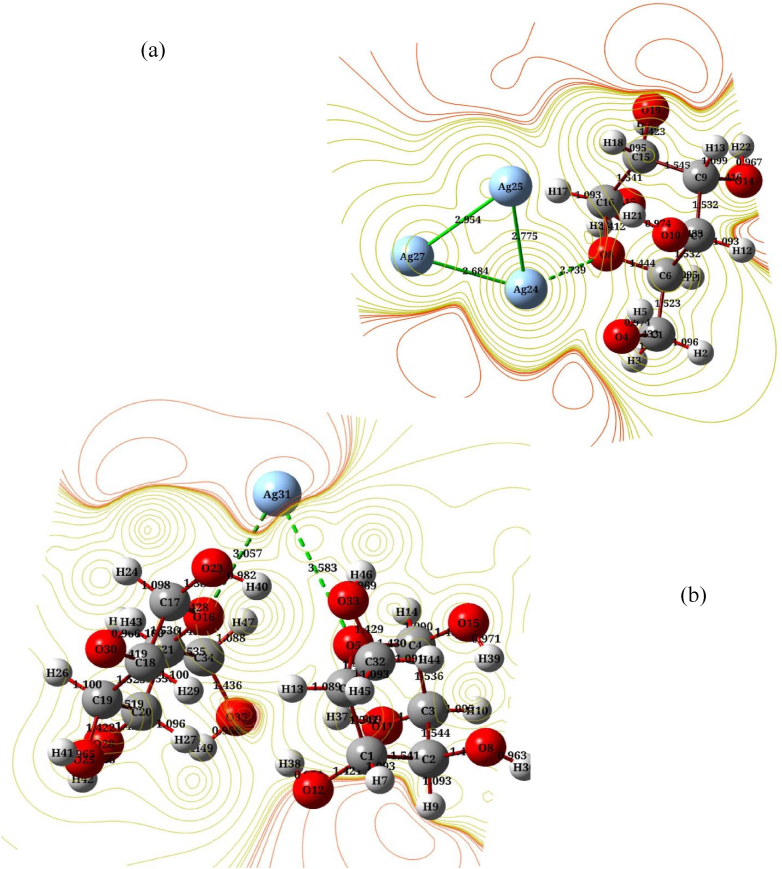
Molecular electrostatic potential (MEP*)* contour surface of (a) α-D glucose with 3 AgNPs and, (b) 2α-D glucose with 1 AgNP.

Fig. 6 (a and b) for the indicated molecules presents how it can easily plot the surfaces of the α-D-glucose with AgNPs molecule to recognize these surfaces. Nevertheless, if isosurface values were plotted for the surfaces of studied structures, only the top surface would be seen.

## 4 Conclusion

Structural and electronic properties of different structures of glucose molecules were investigated after being added to silver nanoparticles. The hybrid function of B3LYP and the basis of 6–311+G∗ set were utilized for the first time to investigate these properties. This project has considered multiple α‐D‐glucose (C_6_H_12_O_6_) molecules adsorbed on an AgNP surface (one Ag atom and a cluster of Ag_3_). The optimized geometries for those configurations were obtained through these theoretical calculations. IR spectra were analyzed to support fingerprints obtained in the range 0–4000 cm^−1^. The results suggest the adsorption of the C-glucose on the AgNPs’ surface due to the interaction between the -COO- functional group of glucose and AgNPs. Remarkable evidence of the AgNPs and α‐D‐glucose interaction was found by the agreement of the comparison with experimental data via the detection of functional group frequencies. Furthermore, it was found that a shift of the peaks towards the short frequencies by replacing three Ag atoms rather than one atom and reducing 2α‐D‐glucose to α‐D‐glucose indicated greater adsorption in the IR because of the interaction of the –COO/Ag.

Moreover, the frontier molecular orbitals were also studied by calculating the energy difference between the HOMO and the LUMO orbitals caused by π →π∗ electronic transitions and illustrated to evaluate the energy gap (E***g***). The energy gap increases from 3.440 to 4.358 eV for C_12_H_24_O_12_Ag and C_6_H_12_O_6_Ag_3_ indicating to the small nanoparticle diameter according to the quantum confinement concept. Hence, glucose molecules act as capping agents for AgNPs to enhance nanoparticle features.

Furthermore, to demonstrate the regions of nucleophilic and electrophilic potential for titled molecules, the diagram of the isosurface and contour were utilized to present diagrams of the molecular electrostatic potential (MEP) and charge densities.

## Additional information

No additional data and information is available for this paper.

## Data availability statement

The authors declare the date is included in the article and no additional data available.

## CRediT authorship contribution statement

**Walaa S. Sarhan:** Writing – original draft, Visualization, Software, Resources, Investigation, Formal analysis, Data curation. **Nagham M. Shiltagh:** Writing – review & editing, Validation, Supervision, Software, Project administration, Methodology, Investigation, Funding acquisition, Data curation, Conceptualization.

## Declaration of competing interest

The authors declare that they have no known competing financial interests or personal relationships that could have appeared to influence the work reported in this paper.
